# Human Chorionic Gonadotropin as a Tumor Marker of Colorectal Cancer: A Case Report in a Non-pregnant Patient

**DOI:** 10.7759/cureus.99480

**Published:** 2025-12-17

**Authors:** Diana Castanheira, Ana Rita Alves, Beatriz O Ferreira, Alexandra Fernandes, Isabel Duarte

**Affiliations:** 1 Obstetrics and Gynecology, Local Health Unit of Leiria Region, Leiria, PRT; 2 Gastroenterology, Local Health Unit of Leiria Region, Leiria, PRT

**Keywords:** alpha-feto protein, colorectal cancer, human chorionic gonadotropin, pregnancy, tumor marker

## Abstract

Tumor markers (TM) are biological substances that, depending on their specificity and sensitivity, can be used for cancer screening, diagnosis, monitoring of disease activity, and assessment of treatment response. Human chorionic gonadotropin (hCG), initially employed in pregnancy tests, is a hormone with diverse physiological functions that also serves as a TM, particularly in trophoblastic and germ cell tumors (GCT), where it contributes to cancer progression.

We report a rare case of rapidly progressing and fatal stage IV colorectal cancer (CRC) based on radiological data in a 41-year-old woman, in whom the typical TM were only slightly elevated or within normal limits. However, serum levels of the free β-subunit of hCG (β‑hCG) and alpha-fetoprotein (AFP) - markers usually associated with GCT - were elevated. Initially, a pregnancy of unknown location (PUL) was suspected, despite a history of definitive contraception and the absence of ultrasound (US) findings suggestive of pregnancy.

Up to 20% of CRCs can secrete β-hCG, usually at low levels, predominantly in tumors located in the rectosigmoid region. β-hCG is physiologically secreted by placental syncytiotrophoblasts during embryo implantation to facilitate trophoblast invasion; analogously, β-hCG-secreting CRC tend to exhibit poor histological differentiation, increased local invasion, and early metastasis, resulting in worse prognosis. In this case, carbohydrate antigen (CA) 19-9 and carcinoembryonic antigen (CEA) were only slightly elevated, and CA 72-4 levels were within the normal range, which is atypical for most CRC. CRC with serum AFP ≥ 200 ng/mL are more likely to present as stage IV disease; however, in this patient, these features were observed at diagnosis despite lower AFP levels. In women of reproductive age, elevated β‑hCG levels can be misleading, as they often suggest pregnancy and may delay accurate diagnosis.

This case highlights a rare presentation of CRC co-expressing β-hCG and AFP. Elevated β-hCG in women of reproductive age can delay recognition of non-gestational malignancies, underscoring the need for careful interpretation in atypical clinical contexts. Co-expression of β-hCG and AFP may indicate a more aggressive tumor phenotype and poorer prognosis. Further studies are warranted to evaluate the potential role of these markers in the diagnosis, monitoring, and prognostication of CRC, particularly in early-onset cases.

## Introduction

Tumor markers (TM) are biological substances produced and released primarily by malignant cells. Depending on their specificity and sensitivity, these molecules serve as indicators of the presence and activity of particular malignancies. Consequently, TM can be used for cancer screening, diagnosis, disease activity monitoring, and assessment of treatment response [1].

Human chorionic gonadotropin (hCG) is a hormone with diverse physiological functions that also acts as a TM, particularly in trophoblastic and germ cell tumors (GCT) of the testis and ovary, where it plays a role in cancer progression, including cell transformation, angiogenesis, metastasis, and immune evasion [1]. This glycoprotein hormone consists of two subunits - alpha (α) and beta (β) - linked noncovalently, and exists in several molecular forms rather than as a single entity: regular hCG, hyperglycosylated hCG, and the free β-subunit of hyperglycosylated hCG (HGfβ) [2,3].

Since its discovery in 1920 and its first application in pregnancy testing, hCG and its molecular variants (total hCG, β-hCG, and others) have found multiple clinical uses, including pregnancy detection and monitoring of early pregnancy progression, aneuploidy screening, detection of pituitary hCG, diagnosis and management of gestational trophoblastic diseases (GTD), quiescent GTD, placental site trophoblastic tumors, testicular GCT, and monitoring of other malignancies [2,4].

Regular hCG is the predominant form during both normal and abnormal pregnancies (such as miscarriage or ectopic pregnancy), hydatidiform moles (HM), and pregnancies composed solely of trophoblastic tissue. During gestation, this hormone plays essential roles in trophoblast differentiation and fetal nutrition by promoting spiral artery angiogenesis within the myometrium. Regular hCG is secreted by fused villous syncytiotrophoblasts and stimulates progesterone production by the corpus luteum in early pregnancy. In contrast, hyperglycosylated hCG is secreted by extravillous invasive cytotrophoblasts (EIC), acting as an autocrine factor that promotes and regulates invasion during implantation, thereby establishing hemochorial placentation. This same mechanism operates in malignancies such as invasive HM and choriocarcinoma, where hyperglycosylated hCG inhibits apoptosis in EIC, enhancing cell invasion, proliferation, and malignant transformation [2,3].

Malignancies of non-trophoblastic origin - including hepatocellular, colorectal, breast, renal, bladder, ovarian, uterine, cervical, brain, and lung cancers - can, in advanced stages, undergo retrograde differentiation and produce HGfβ. This molecule functions as an autocrine factor that inhibits apoptosis, promoting cancer cell growth and proliferation [2,5,6]. Although HGfβ is a nonspecific and unreliable TM for non-trophoblastic malignancies, its presence is strongly associated with poor prognosis. The β-subunit core fragment, the final degradation product of HGfβ, can be detected in urine and represents a more sensitive TM. Therefore, urinary β-subunit core fragment testing may have potential as a general cancer screening tool; however, due to its lack of specificity, a positive result would necessitate a whole-body evaluation to identify the primary tumor site [2].

This case report describes a non-trophoblastic malignancy associated with elevated serum β-hCG levels in a woman of reproductive age.

## Case presentation

A 41-year-old woman with a history of chronic hypertension and three previous cesarean deliveries, with tubal ligation performed during the last procedure, presented to our emergency department with suprapubic abdominal pain that had begun four days earlier. She had no prior or current gastrointestinal or gynecological complaints and denied recent weight loss. Her last menstrual period had occurred 12 days earlier, with previously regular cycles.

Initially, given her symptoms, she was evaluated in the general surgery emergency unit. Abdominal US revealed thickening and hypoechogenicity of the sigmoid colon with diverticula, suggesting possible diverticulitis. However, she was referred to the gynecology emergency department due to an incidental finding of elevated serum β-hCG levels (261.4 mIU/mL; normal range for nonpregnant woman is 1.1-2.9mIU/mL). Pelvic examination and US were unremarkable, with no evidence of acute gynecological pathology.

**Figure 1:**
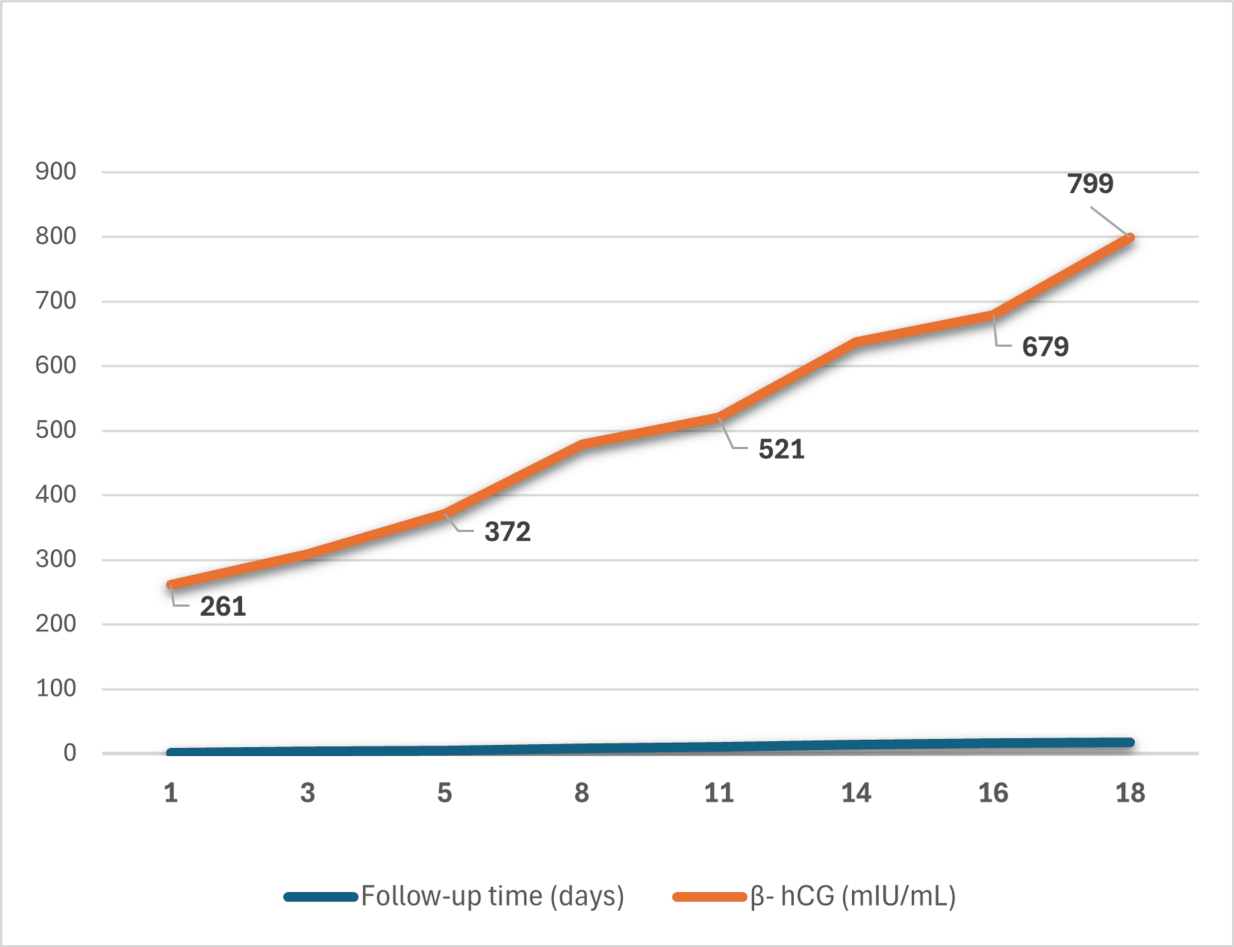
Serum β-human chorionic gonadotropin (hCG) levels Trend of serum β-hCG levels over time during the initial follow-up period.

**Figure 2:**
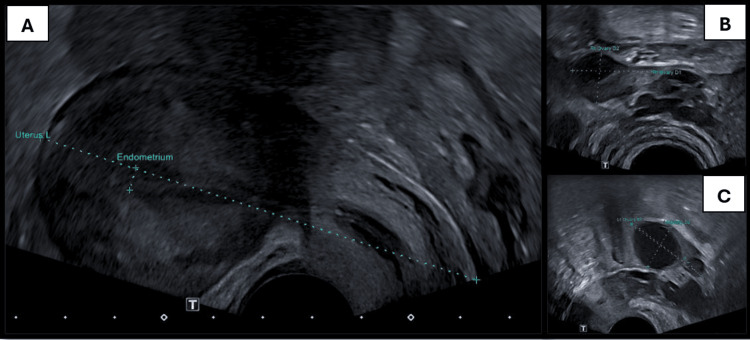
Pelvic US images Pelvic US images showing an empty uterine cavity and an isthmic notch consistent with prior cesarean sections (A); normal appearance of the right and left adnexal regions (B and C, respectively), with ovaries demonstrating follicular activity. No pelvic or abdominal free fluid was observed.

A diagnostic hypothesis of pregnancy of unknown location (PUL) was proposed, despite the prior tubal ligation. The patient was subsequently kept under clinical, analytical, and imaging surveillance, with serial measurements of serum β-hCG levels (Figure 1) and pelvic US examinations, according to established protocols [7,8]. In all assessments, pelvic US didn’t reveal findings compatible with intrauterine or ectopic pregnancy (Figure 2).

**Figure 3:**
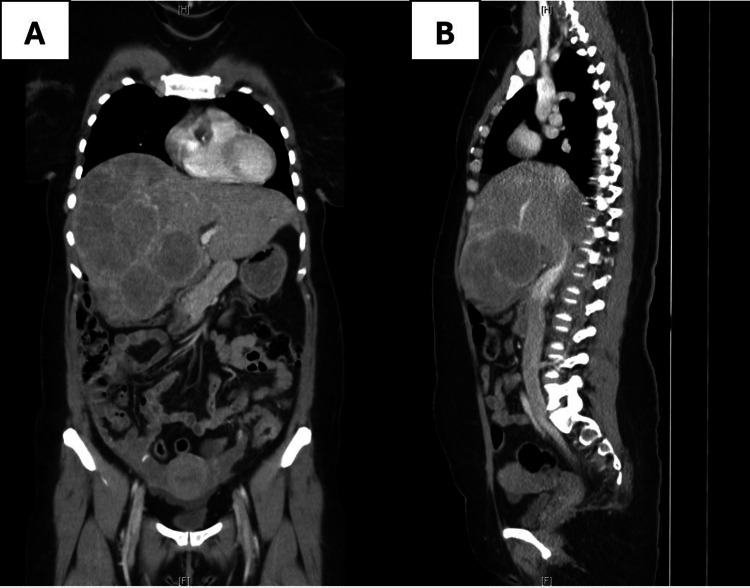
CT scan images Contrast-enhanced abdominopelvic CT scan demonstrating multiple solid hepatic nodules, highly suggestive of metastatic disease. A - coronal view. B - sagital view.

During follow-up, the patient experienced a brief episode of scant metrorrhagia on the second day. By the 13th day, she reported constant right upper quadrant pain, constipation, and a first episode of rectal bleeding. On examination, jaundice was observed. Abdominopelvic computed tomography (CT) revealed hepatomegaly with multiple solid hypodense nodules, the largest conglomerate measuring 13 cm in diameter, consistent with metastatic lesions (Figure 3). In addition, millimetric lymphadenopathies adjacent to the descending colon and circumferential thickening of the colonic wall were identified.

Serum TM testing showed slightly elevated carcinoembryonic antigen (CEA) (5.5 ng/mL; normal if < 3.0 ng/mL) and carbohydrate antigen (CA) 19-9 (37.0 U/mL; normal if < 35.0 U/mL). Alpha-fetoprotein (AFP) was more markedly elevated (34.7 ng/mL; normal if <9.0ng/mL). Other TM, including CA 72.4 (2.2UI/mL; normal if <6.9UI/mL) and CA 125 (16.3U/mL; normal if <35U/mL), were within normal limits.

**Figure 4:**
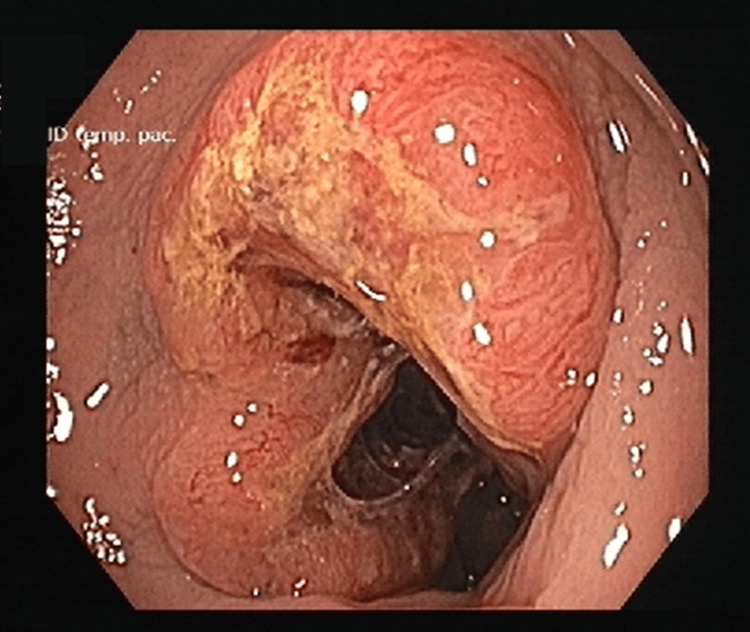
Colonoscopic photography Colonoscopic view showing an almost circumferential ulcerovegetative lesion located about 25 cm from the anal verge. Histological analysis revealed a low-grade invasive colorectal adenocarcinoma.

**Figure 5:**
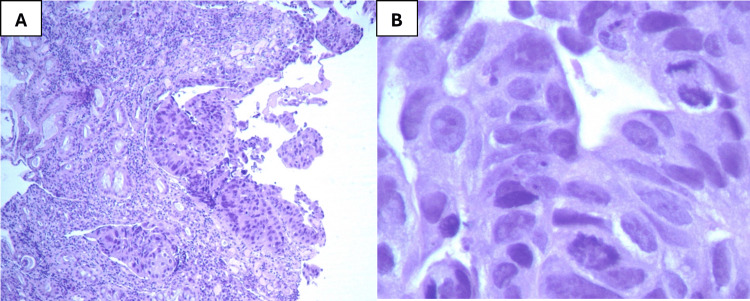
Histology analysis images Histology analysis showing a low-grade invasive colorectal adenocarcinoma (hematoxylin and eosin staining; magnification: A - 40x and B - 400x).

On the 23rd day of follow-up, colonoscopy revealed an almost circumferential ulcerovegetative lesion located approximately 25 cm from the anal verge (Figure 4), which was biopsied. Histopathological examination confirmed a low-grade invasive colorectal adenocarcinoma (Figure 5). The patient was subsequently referred to the general surgery department for therapeutic management.

On the 30th day of follow-up, she returned to the emergency department with worsening abdominal pain, rectal bleeding, and jaundice. She was hemodynamically stable, but laboratory testing revealed anemia (hemoglobin 7.3 g/dL). The patient received a blood transfusion and was discharged.

Five days later (day 35 of follow-up), she returned due to clinical deterioration, presenting with abdominal pain and biliary vomiting that had begun five hours earlier. Shortly after admission, she experienced a cardiorespiratory arrest, which could not be reversed despite resuscitation efforts.

## Discussion

This case describes a young adult woman with colorectal adenocarcinoma and elevated serum β-hCG levels within a range typically suggestive of pregnancy, which could occur either in isolation or concurrently with CRC. Initially, the main concern was an ectopic pregnancy, as intrauterine implantation was unlikely due to prior tubal ligation. However, during follow-up, no imaging evidence of pregnancy was identified.

It has been known for over three decades that up to 20% of CRC can secrete β-hCG, usually at low levels, and that most of these tumors are located in the rectosigmoid region (approximately 90%) [3,5]. β-hCG is physiologically secreted by placental syncytiotrophoblasts during embryo implantation to facilitate trophoblastic invasion. Analogously, β-hCG-secreting CRC tend to exhibit poor histological differentiation, greater local invasion, and increased metastatic potential (especially to lymph nodes and the liver), leading to a worse prognosis [3,6]. Unlike CEA, which typically rises in advanced stages, β-hCG may increase in early-stage disease, and its expression can help predict recurrence risk following initial treatment [6].

Based on its association with deeper invasion into the colonic muscular layer, Jiali Li et al. demonstrated that β-hCG expression may serve as a potential marker for detecting early invasion and predicting poorer prognosis in CRC [6]. Furthermore, in addition to serum β-hCG measurement, immunohistochemical detection of β-hCG in biopsy or surgical specimens has also been linked to more aggressive tumor behavior, although serum levels appear to have greater predictive value than tissue staining [5,6,9,10].

Other TM associated with poor prognosis in CRC in both males and females include CA 19-9, CA 242, and CA 72-4 [9]. In the present case, CEA and CA 19-9 were only slightly elevated, and CA 72-4 remained within normal limits - an atypical finding for most CRC.

Few cases of β-hCG-secreting CRC have been reported in young adult patients [9,11]. In women of reproductive age, elevated β-hCG levels present a diagnostic challenge, as they raise suspicion of PUL or trophoblastic disease, such as invasive HM [9,12]. In this case, pelvic US showed no evidence of intrauterine or ectopic pregnancy, and serum β-hCG concentrations were far below the levels typically seen in GTD, making this diagnosis unlikely [13]. Uterine evacuation was not indicated because pregnancy was improbable given the patient’s previous tubal ligation. However, due to the possibility of PUL, methotrexate therapy could have been considered [7,8].

β-hCG-secreting CRC are associated with lower survival rates and increased propensity for local invasion and metastasis compared with non-secreting tumors [6]. There is also a possibility of metastatic dedifferentiation of CRC into GCT-like morphology, leading to co-secretion of β-hCG and AFP [11]. Similar to trophoblastic tumors, β-hCG-secreting malignancies may demonstrate high chemosensitivity, with serum β-hCG levels decreasing markedly following treatment [9,13,14]. Thus, serial measurement of serum β-hCG can provide valuable information regarding treatment response, disease recurrence, and progression risk in CRC [6,9]. In this case, however, due to the extremely rapid disease progression and fatal outcome, treatment response monitoring was not possible.

AFP is a serum glycoprotein synthesized during fetal development by the liver and yolk sac and is rapidly suppressed after birth [15]. Elevated AFP levels in adults are typically associated with hepatocellular carcinoma or yolk sac tumors, and secretion by CRC is rare, with only a few cases reported since the first description in 1985 [15,16]. AFP-producing gastrointestinal tumors may arise throughout the digestive tract but occur most frequently in the stomach (>80%) [15,17]. These AFP-producing CRC often present with advanced disease at diagnosis, displaying a high rate of liver metastasis (up to 25%), poor differentiation (50%), deep local invasion (80%), lymph node metastasis (up to 60%), and poor prognosis [16-18]. Kong et al. reported that CRC with serum AFP ≥ 200 ng/mL are more likely to present with stage IV disease and liver metastasis compared with cases showing lower levels [17]. In the present case, these features were already evident at diagnosis, despite lower AFP levels (34.7 ng/mL). Since AFP was assessed only once, its evolution over time and potential response to treatment could not be evaluated.

The incidence of CRC has been increasing over recent decades, likely due to early-life or young-adult exposure to risk factors such as obesity, physical inactivity, and antibiotic-induced alterations in the gut microbiome. CRC currently ranks as the third most common cancer in men and women (10.4% and 8.9%, respectively) and is the second leading cause of cancer-related death worldwide, according to GLOBOCAN 2022 estimates [19]. Advancing age remains the major risk factor, with over 90% of cases occurring in individuals older than 50 years, and incidence is generally higher in men than in women [20].

Early-onset colorectal cancer (EOCRC), defined as CRC diagnosed before age 50, as in our patient, has shown an alarming increase in both developed and developing countries, despite an overall decline in CRC incidence in high-income nations [20]. Several factors have been implicated in this trend, including genetic predisposition, environmental and lifestyle factors (such as metabolic syndrome, alcohol consumption, ulcerative colitis, physical inactivity, low vitamin D intake, high red meat and sugar-sweetened beverage consumption), and alterations in the gut microbiota [20].​​​​​​​ Although hereditary CRC accounts for only about 5% of all cases, inherited syndromes are found in a higher proportion (up to 16%) among early-onset cases [20].​​​​​​​ Germline mutations in the APC gene (familial adenomatous polyposis) and deoxyribonucleic acid (DNA) mismatch repair genes (Lynch syndrome) are the most commonly identified. Recent genome-wide association studies have revealed additional genetic variants, underscoring the need for further investigation to clarify their clinical implications in CRC epidemiology [20].

## Conclusions

The co-expression of β-hCG and AFP in patients with colorectal adenocarcinoma is an exceptionally rare association, not previously described, and appears to correlate with poor prognosis, increased local invasion, higher metastatic potential, and reduced survival. Nevertheless, large-scale studies are required to determine whether systematic serum measurement and/or immunohistochemical detection of β-hCG and AFP could provide prognostic or therapeutic value for patients with CRC, particularly in early-onset cases. 

In women of reproductive age, elevated serum β-hCG levels can be misleading, as they often prompt the immediate consideration of pregnancy and may delay the diagnosis of other conditions, particularly malignancies. Therefore, in women using highly effective, non-user-dependent contraceptive methods, β-hCG should not be regarded solely as a pregnancy marker but also as a potential TM when clinical or imaging findings are inconsistent with pregnancy.
